# Pervasive survival of expressed mitochondrial *rps14 *pseudogenes in grasses and their relatives for 80 million years following three functional transfers to the nucleus

**DOI:** 10.1186/1471-2148-6-55

**Published:** 2006-07-14

**Authors:** Han Chuan Ong, Jeffrey D Palmer

**Affiliations:** 1 Department of Biology, Indiana University, Bloomington, IN 47405, USA

## Abstract

**Background:**

Many mitochondrial genes, especially ribosomal protein genes, have been frequently transferred as functional entities to the nucleus during plant evolution, often by an RNA-mediated process. A notable case of transfer involves the *rps14 *gene of three grasses (rice, maize, and wheat), which has been relocated to the intron of the nuclear *sdh2 *gene and which is expressed and targeted to the mitochondrion via alternative splicing and usage of the *sdh2 *targeting peptide. Although this transfer occurred at least 50 million years ago, i.e., in a common ancestor of these three grasses, it is striking that expressed, nearly intact pseudogenes of *rps14 *are retained in the mitochondrial genomes of both rice and wheat. To determine how ancient this transfer is, the extent to which mitochondrial *rps14 *has been retained and is expressed in grasses, and whether other transfers of *rps14 *have occurred in grasses and their relatives, we investigated the structure, expression, and phylogeny of mitochondrial and nuclear *rps14 *genes from 32 additional genera of grasses and from 9 other members of the Poales.

**Results:**

Filter hybridization experiments showed that *rps14 *sequences are present in the mitochondrial genomes of all examined Poales except for members of the grass subfamily Panicoideae (to which maize belongs). However, PCR amplification and sequencing revealed that the mitochondrial *rps14 *genes of all examined grasses (Poaceae), Cyperaceae, and Joinvilleaceae are pseudogenes, with all those from the Poaceae sharing two 4-NT frameshift deletions and all those from the Cyperaceae sharing a 5-NT insertion (only one member of the Joinvilleaceae was examined). cDNA analysis showed that all mitochondrial pseudogenes examined (from all three families) are transcribed, that most are RNA edited, and that surprisingly many of the edits are reverse (U→C) edits. Putatively nuclear copies of *rps14 *were isolated from one to several members of each of these three Poales families. Multiple lines of evidence indicate that the nuclear genes are probably the products of three independent transfers.

**Conclusion:**

The *rps14 *gene has, most likely, been functionally transferred from the mitochondrion to the nucleus at least three times during the evolution of the Poales. The transfers in Cyperaceae and Poaceae are relatively ancient, occurring in the common ancestor of each family, roughly 80 million years ago, whereas the putative Joinvilleaceae transfer may be the most recent case of functional organelle-to-nucleus transfer yet described in any organism. Remarkably, nearly intact and expressed pseudogenes of *rps14 *have persisted in the mitochondrial genomes of most lineages of Poaceae and Cyperaceae despite the antiquity of the transfers and of the frameshift and RNA editing mutations that mark the mitochondrial genes as pseudogenes. Such long-term, nearly pervasive survival of expressed, apparent pseudogenes is to our knowledge unparalleled in any genome. Such survival probably reflects a combination of factors, including the short length of *rps14*, its location immediately downstream of *rpl5 *in most plants, and low rates of nucleotide substitutions and indels in plant mitochondrial DNAs. Their survival also raises the possibility that these *rps14 *sequences may not actually be pseudogenes despite their appearance as such. Overall, these findings indicate that intracellular gene transfer may occur even more frequently in angiosperms than already recognized and that pseudogenes in plant mitochondrial genomes can be surprisingly resistant to forces that lead to gene loss and inactivation.

## Background

Once a free-living α-proteobacterium, the mitochondrion has experienced numerous gene losses over time as part of its endosymbiotic lifestyle, leading to a greatly reduced number and diversity of genes [1]. Although functional translocation of coding sequences from the mitochondrion to the nucleus has essentially ceased in animal mitochondria, these seemingly difficult events occur at surprisingly high frequency in flowering plants. Greatly extending findings from past studies focusing on single genes in a limited group of angiosperms (reviewed in [1-3]), Adams et al. [4] surveyed 280 diverse angiosperms by Southern blot hybridization and found that all 14 ribosomal protein genes and both *sdh *genes had been frequently lost from the mitochondrial genome and, most likely, functionally transferred to the nucleus, whereas only two putative losses were detected among the other 24 genes, most of which encode respiratory proteins. These differences in frequency of functional transfer to the nucleus (and associated mitochondrial gene loss) are probably largely due to relative ease of protein import back into the mitochondrion [5-7].

One of the most well-studied and interesting of the frequently transferred ribosomal protein genes is *rps14*, which was estimated to have been lost from the mitochondrial genome 27 times separately among the 280 angiosperms surveyed by Adams et al. [4]. Mitochondrial genome sequencing has confirmed two of these inferred losses, in *Zea *[8] (also see [9]) and in *Beta *[10], but not a third, in *Nicotiana*, where a pseudogene copy comprising most of *rps14 *is instead present in the mitochondrial genome [11] (see Discussion for details). *rps14 *is also found only as a pseudogene in the mitochondrial genome in *Arabidopsis *[12-14], potato [15], *Prunus *[16], cucumber [17], rice [18,19], and wheat [20]. So far, an intact and potentially functional mitochondrial copy of *rps14 *has been identified by sequencing in relatively few plants, namely, *Oenothera *[21], *Brassica *[22,23], pea [24], and broad bean [25].

Transferred, functional copies of *rps14 *have been well characterized from the nuclear genomes of *Arabidopsis *[26] and three grasses (rice [18], maize [9,27], and wheat [20]), while a less well- characterized but likely functional and transferred *rps14 *gene is represented in EST collections of tomato. The nuclear *rps14 *genes of rice, maize, and wheat unquestionably result from the same transfer event, as they share a highly derived and fascinating structure acquired post-transfer and described below. In contrast, the different structures of nuclear *rps14 *(in particular, with respect to their N-terminal targeting elements acquired post transfer) in *Arabidopsis *and tomato relative to one another and the grasses indicate that they are probably each the product of evolutionarily independent transfer events.

The nuclear *rps14 *genes of the three grasses are located within the intron of an anciently transferred nuclear gene that encodes mitochondrial succinate dehydrogenase subunit 2 (SDH2). Alternate splicing of the *sdh2*-*rps14 *primary transcript results in two different products, a conventional *sdh2 *mRNA containing both *sdh2 *exons and a chimeric mRNA containing the first exon of *sdh2 *and *rps14 *[9,18]. The products of both mRNAs are thought to be targeted to the mitochondrion using the N-terminal, cleavable extension of SDH2, followed by proteolytic processing of the SDH2-RPS14 fusion precursor to generate mature RPS14 [27].

The mitochondrion-to-nucleus transfer of *rps14 *in grasses must have occurred at least 50 million years ago, i.e., in the last common ancestor of rice, maize, and wheat. It is therefore striking that nearly intact – and expressed – copies of this gene are still present in the mitochondrial genomes of two of these plants, rice [18] and wheat [20] (as noted above, *rps14 *is absent from maize mitochondrial DNA). To further explore this situation, including determining exactly when the grass *rps14 *transfer occurred and whether other transfers of this gene have occurred in related plants, we have examined the structure and expression of *rps14 *from a large number of grasses and other members of the Poales. Our results show that the grass transfer occurred roughly 80 million years ago, in the common ancestor of all grasses, and is accompanied by widespread, persistent retention of expressed and nearly intact pseudogenes among many diverse grass lineages. We also report two new, apparent cases of *rps14 *transfer in Poales, one relatively ancient and the other more recent, both of which are also accompanied by retention of expressed mitochondrial pseudogenes.

## Results

### Slot-blot survey for presence/absence of *rps14 *in mtDNA

**Figure 1:**
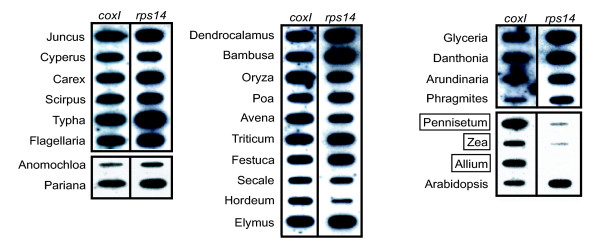
**Slot blot survey for the presence or absence of *rps14 *in the mitochondrial genome of members of the Poales**. Shown are 24 of 38 Poales taxa examined by slot blot hybridization with mt *cox1 *and *rps14 *probes from *Oryza*. The three boxed taxa have highly reduced hybridization of *rps14 *relative to *cox1*, suggesting that most of all of *rps14 *has been lost from the mitochondrial genome. Positive (*Arabidopsis*) and negative (*Allium*) controls were chosen based on the results of Adams et al. [4]. The two probes were hybridized sequentially to the same membrane, which was stripped in 0.1 × SSC at 100°C after the first hybridization.

**Figure 2:**
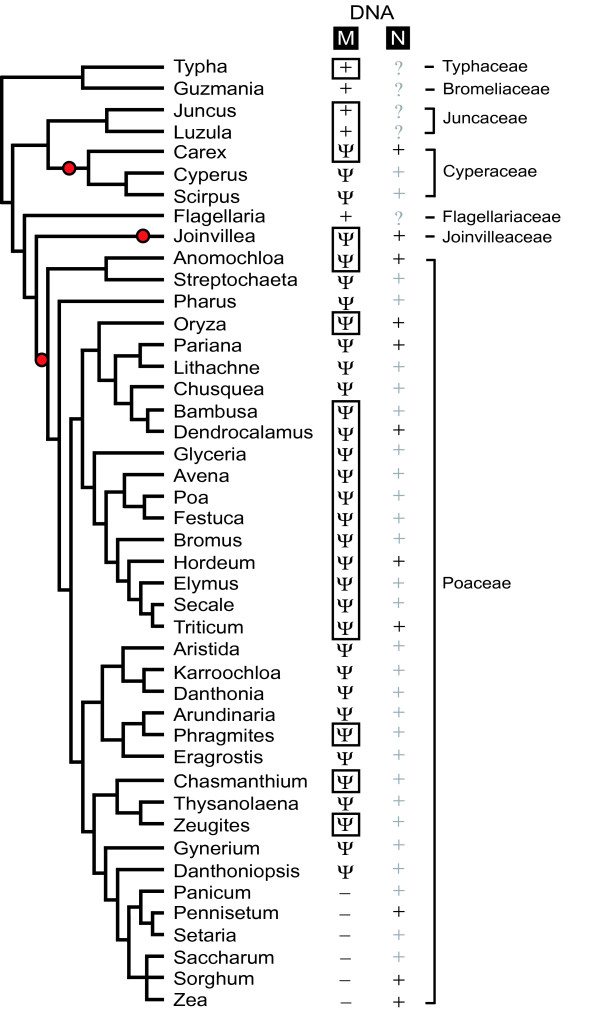
***rps14 *gene status in 44 genera of Poales**. The status of *rps14 *in either the mitochondrial (M) or nuclear (N) genomes is marked on a phylogenetic tree of Poales as follows: A dark "+" indicates that *rps14 *is an intact open reading frame over the length of the gene sequenced. A light "+" indicates that a nuclear *rps14 *sequence was not obtained by PCR, but is inferred to be present based on the absence of an intact *rps14 *gene in the mitochondrial genome of that species. A "ψ" indicates that only a pseudogene form of *rps14 *is present in the mitochondrial genome. A "-" indicates that *rps14 *is inferred to be absent from the mitochondrial genome based on negative slot blot and/or PCR results. A question mark indicates uncertainty as to whether an intact copy of *rps14 *is present in the nucleus because a functional copy was recovered from the mitochondrial genome and because repeated PCR attempts failed to yield a putative nuclear *rps14 *sequence. Red bullets mark three separate transfers of *rps14 *to the nucleus. The *Joinvillea *transfer is shown as very recent because of the nuclear gene's limited divergence (see text, Figure 3, and Additional File 1), whereas the other two transfers are arbitrarily positioned at the midpoint of their internodes. Black boxes mark mitochondrial sequences for which RNA editing information has been obtained (Table 1). The estimate of Poales phylogeny are based on GPWG (2001) [61], Kellogg (2001) [62], and Bremer (2002) [38].

We conducted DNA slot blot hybridizations to survey for the presence/absence of *rps14 *in the mitochondrial genomes of 38 Poales taxa (24 of which are shown in Figure 1). As a positive control, the membranes were first probed for mitochondrial *cox1*, a gene found universally in mitochondrial genomes of all examined eukaryotes, including the 280 angiosperms surveyed by Adams et al. [4]. As expected, the *cox1 *probe hybridized well to all Poales DNAs tested. The membranes were then stripped and re-hybridized with a mitochondrial *rps14 *gene probe from *Oryza*. This resulted in strong hybridization, roughly proportional from slot to slot compared to the *cox1 *control, to all Poales taxa surveyed with the exceptions of *Pennisetum *and *Zea *(Figure 1). These slot blot results are consistent with published Southern blot data for eight of these same Poales taxa (Adams et al. [4], as well as the complete mitochondrial genome sequence of *Zea *[8], which shows complete absence of *rps14*. The two taxa showing little *rps14 *hybridization form a monophyletic group (corresponding to subfamily Panicoideae) relative to the other Poales examined (Figure 2). Therefore, it would appear that *rps14 *has been lost from the mitochondrial genome just once among the Poales examined, in the common ancestor of the Panicoideae.

We attribute the weak *rps14 *signal in the *Pennisetum *and *Zea *slots to either non-specific background noise in the slot or to hybridization to the transferred nuclear copy of the gene, which exists at low copy number compared to the mitochondrial genome (all DNAs tested were total genomic DNAs). *Hordeum *also showed reduced hybridization strength with *rps14 *(Figure 1), suggesting that much or all of its gene might have been lost from the mitochondrial genome. However, as described below, we succeeded in isolating by PCR a nearly full-length copy of *rps14 *from *Hordeum *with undiverged sequences typical of mitochondrial *rps14 *and, more importantly, we isolated *Hordeum rps14 *cDNA sequences that were identical to the PCR sequence except for six sites of mitochondrial-diagnostic RNA editing. Therefore, we are confident that *Hordeum *mtDNA does contain *rps14*. We attribute its reduced slot blot hybridization to, most likely, slot-specific DNA shedding after the initial *cox1 *hybridization. Alternatively, the reduced intensity of mitochondrial *rps14 *relative to *cox1 *in *Hordeum *could be real, if the former gene were located only on a low-copy-number form of the genome (a "sublimon"), with *cox1 *present on a standard, high-copy-number genome conformation.

### Isolation and sequencing of mitochondrial *rps14 *genes and cDNAs

Both previously sequenced mitochondrial *rps14 *genes from grasses (*Oryza *and *Triticum*) are pseudogenes [18,20]. To determine whether the many other strongly hybridizing mitochondrial *rps14 *sequences from grasses are also pseudogenes, or whether some might be still intact and possibly functional, we used degenerate primers to PCR-amplify and then sequence *rps14 *genes from these and various other Poales. No mitochondrial-like *rps14 *sequence was isolated from the two panicoid grasses examined by slot blots (Figure 1) or from the four other panicoids examined by PCR (*Panicum*, *Setaria*, *Saccharum *and *Sorghum*). This further supports the idea that the gene disappeared from the mitochondrial genome in the common ancestor of the Panicoideae. In contrast, intact or nearly intact copies of mitochondrial-like *rps14 *were isolated from the other 38 Poales taxa examined.

**Figure 3:**
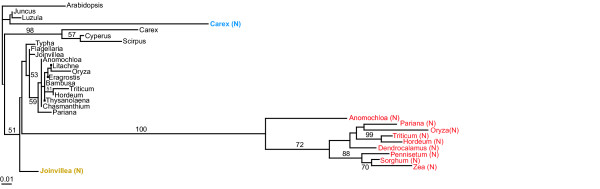
**Phylogenetic analysis of mitochondrial and nuclear *rps14 *sequences**. Maximum likelihood tree of *rps14 *nucleotide sequences putatively located in the mitochondrial genome (shown in black) and nuclear genome [color-coded according to Poales families: red (Poaceae), orange (Joinvilleaceae), and blue (Cyperaceae)]. Bootstrap support values above 50% are shown. Scale bar at bottom indicates number of substitutions per site.

*rps14 *sequences were deemed to be of likely mitochondrial provenance if one or both of the following criteria were met: 1) they aligned well with the published mitochondrial *rps14 *sequences of *Oryza *and *Triticum *with limited divergence (Figure 3; Additional File 1), and 2) they were determined to undergo mitochondrial-characteristic/diagnostic C→U and/or U→C RNA editing. The latter provides strong evidence for mitochondrial provenance (RNA editing is unknown for plant nuclear genes). *rps14 *cDNA sequences are already available for *Oryza *[18] and *Triticum *[20] and were determined for 19 additional putative mitochondrial genes. Of these 21 genes, 15 showed evidence of mitochondrial-characteristic RNA editing (Table 1; see Methods for all controls and precautions taken to ensure that the inferred RNA edits are all real). The first criterion is premised on the fact that in virtually all plants, rates of nucleotide substitutions are much lower (typically 10–20 times lower at synonymous sites) for mitochondrial genes than for nuclear genes [28-30]. Consistent with this, in all examined cases, plant mitochondrial genes transferred to the nucleus have accumulated much more sequence divergence than their homologs still present in the mitochondrion (e.g., [30,31]). Although the pseudogene sequences from *Cyperus *and *Scirpus *were not checked for editing status and are relatively divergent (Figure 3; Additional File 1) we nonetheless are able to assign them to the mitochondrial genome because of their close relationship (including one shared frameshift; Figure 3 and Additional File 1) with a third pseudogene sequence from the Cyperaceae, from *Carex*, for which RNA editing *was *demonstrated (Table 1).

**Table 1:** RNA editing of mitochondrial *rps14 *genes and pseudogenes in Poales

Plants	Total edits	C-to-U editing	U-to-C editing	Clones sampled	Clones edited^a^
*Typha*	3	2	1	4	4
*Juncus*	1	1	0	23	1
*Luzula*	0	0	0	20	0
*Carex*ψ	1	1	0	2	2
*Joinvillea*ψ	2	1	1	7	7
*Anomochloa*ψ	2	1	1	4	4
^b^*Oryza*ψ	0	0	0	N/A	N/A
*Bambusa*ψ	1	0	1	4	1
*Dendrocalamus*ψ	1	1	0	4	3
*Glyceria*ψ	0	0	0	8	0
*Avena*ψ	1	1	0	3	1
*Poa*ψ	0	0	0	3	0
*Festuca*ψ	2	2	0	5	2
*Bromus*ψ	0	0	0	2	0
*Hordeum*ψ	6	4	2	5	3
*Elymus*ψ	3	2	1	7	3
*Secale*ψ	1	0	1	4	1
^c^*Triticum*ψ	2	2	0	14	10
*Phragmites*ψ	0	0	0	4	0
*Chasmanthium*ψ	1	0	1	2	2
*Zeugites*ψ	1	0	1	4	2

N/A = Data not available

^a ^No partial editing was observed; i.e., whenever a cDNA clone had any edits, it had *all *the edits found in aggregate among all cDNAs sequenced for that gene.

^b ^Kubo et al. [18]

^c ^Sandoval et al. [20]

All 29 Poaceae mitochondrial *rps14 *sequences isolated are pseudogenes sharing the same two frameshift deletions; these are both 4 NT in length and are only 8 NT apart (Additional File 1). These two deletions are estimated to have occurred about 80 million years ago, in the last common ancestor of all grasses (Figure 2). Of the nine non-grass Poales examined, five (representing four families) contain intact mitochondrial *rps14 *genes, whereas all three Cyperaceae and the one Joinvilleaceae examined possess pseudogenes of *rps14 *in their mitochondrial genomes (Figure 2; Additional File 1). *Joinvillea *contains a single frameshift, which is possibly shared with one of the two grass-wide 4-NT deletions (see end of Results), whereas there are between three and five frameshifts (including one shared by all three genes) in each Cyperaceae mitochondrial gene (Additional File 1).

### Isolation and sequencing of nuclear *rps14 *genes

We also wished to amplify any nuclear copies of *rps14 *present in the Poales, but the much higher substitution rates and accumulated divergence in the nucleus combined with the much lower copy number of the nuclear genome made this more challenging than amplification of mitochondrial *rps14*. Nuclear copies of *rps14 *have already been reported from three grasses (*Zea*, *Oryza*, and *Triticum*; [9,18,20]). After numerous attempts with repeated adjustments to thermocycling parameters and primer design, we were able to amplify putative nuclear copies of *rps14 *sequence from 8 additional Poales (Figure 2). All of these putative nuclear *rps14 *genes have intact open reading frames, and in all cases, *rps14 *is either a pseudogene in the mitochondrial genome (*Carex*, *Joinvillea*, *Anomochloa*, *Pariana*, *Dendrocalamus*, *Hordeum*), as is also the case in *Oryza *and *Triticum*, or is entirely absent from the mitochondrial genome (*Pennisetum*, *Sorghum*), as in *Zea*. We infer [light pluses in the nuclear (N) column in Figure 2] that an intact, functional copy of *rps14 *exists in those many grasses and two Cyperaceae for which mitochondrial *rps14 *is a pseudogene and for which a second, putative nuclear copy was not isolated. In this respect, note that RPS14 is an essential protein in the mitochondrial ribosome, at least in yeast where this issue has been examined [32].

**Figure 4:**
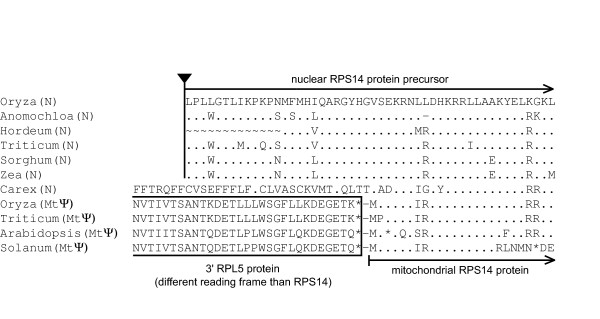
**Sequences upstream of *rps14 *consistent with separate transfers in Cyperaceae and Poaceae**. Shown is an amino acid alignment of the 5' end of RPS14 and upstream sequences. Sequences are aligned relative to *Oryza *nuclear *rps14 *with identical amino acids indicated by dots, gaps by dashes, missing data by "~", and intron-exon junction by a triangle. The lack of homology between the putative targeting peptides (the N-terminal extensions past the methionine start of the mitochondrial *rps14 *genes) of the *Carex *and grass nuclear genes is consistent with their being the result of separate functional transfers. Except for a methionine so close (7 amino acids) to the start of the mature RPS14 protein as to produce a potential targeting peptide too short to likely be functional, the *Carex *gene lacks a potential (methionine) start codon in the region for which sequence has been obtained.

Nuclear assignment of the newly isolated, intact *rps14 *genes from *Anomochloa*, *Pariana*, *Dendrocalamus*, *Hordeum*, *Pennisetum*, and *Sorghum *is straightforward. This is because all six sequences are highly divergent from mitochondrial *rps14 *genes and group with 100% bootstrap support with the previously characterized nuclear *rps14 *genes from *Oryza*, *Zea*, and *Triticum *(Figure 3). Moreover the branching order among these nine *rps14 *genes (Figure 3) is congruent with organismal phylogeny (Figure 2). Finally, we determined (Figure 4 and data not shown) that three of the six newly characterized genes (from *Hordeum*, *Sorghum*, and, importantly, the basal grass *Anomochloa*) have both homologous N-terminal targeting sequences and the same nuclear genomic location (inserted within the spliceosomal intron of nuclear *sdh2*) as previously shown for nuclear *rps14 *of *Oryza*, *Zea*, and *Triticum *[9,18,20]. We therefore conclude that the functional transfer of *rps14 *from the mitochondrial genome into the nuclear *sdh2 *intron occurred in a common ancestor of all grasses.

**Figure 5:**
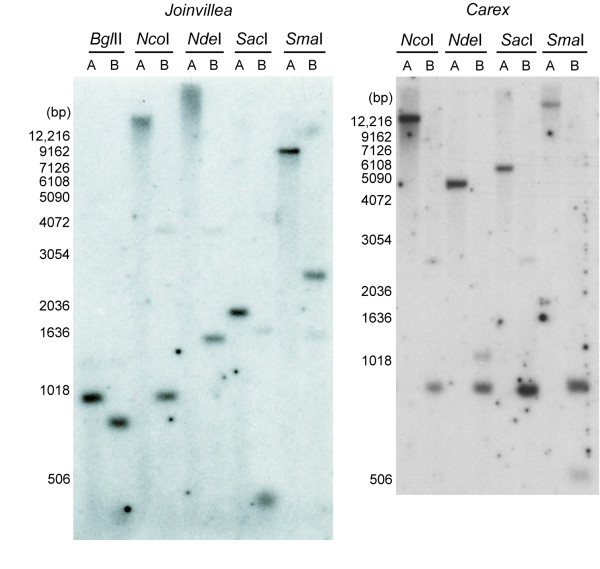
**Southern blot hybridization indicating that *Joinvillea *and *Carex *mtDNAs contain only one copy of *rps14***. The probe used in both panels contains the entire (~280 bp) mitochondrial *rps14 *gene from *Oryza*. The A lanes contain single digests with the indicated restriction enzymes, each set of which does not cut within the mitochondrial *rps14 *pseudogene of *Joinvillea *or *Carex*, respectively. The B lanes are double digests with the indicated enzyme plus *Hind *III, which cuts each mitochondrial *rps14 *locus 70 bp from its 5' end, but which does not cut the putatively nuclear *rps14 *genes from *Joinvillea *and *Carex*.

Unlike the grass situation, assignment of the second, intact copy of *rps14 *from *Carex *as nuclear in location was not possible based on differential sequence divergence. This is because this sequence is not significantly more divergent (Figure 3) than the pseudogene copy of *rps14 *that is assigned to the mitochondrial genome based on its RNA editing status (Table 1). In fact both genes from *Carex *are relatively divergent compared to most mitochondrial *rps14 *genes and pseudogenes (Figure 3; Additional File 1). Southern blot hybridization was performed to assess whether one or both copies of *rps14 *are present in *Carex *mtDNA. A nearly full-length (280 bp) *rps14 *gene segment from *Oryza *mtDNA was used as the probe, as this sequence is roughly equally divergent from both *Carex *genes. The *Oryza *gene hybridized to only a single band in all four single digests of *Carex *total DNA (Figure 5). Double digestion with each of the same four enzymes plus *Hind *III gave the pattern expected for a mitochondrial location of the *rps14 *pseudogene that was already assigned to the mitochondrial genome based on its RNA editing status. That is, *Hind *III doesn't cut the putative nuclear *rps14 *gene of *Carex*, but does cut the mitochondrial gene 70 NT from its 5' end, and the double digests consistently produced two bands of the relative intensities expected given the location of this *Hind *III site (Figure 5). There is a formal possibility that the second, putatively nuclear *rps14 *gene of *Carex *is located instead in the mitochondrial genome, immediately downstream of the mitochondrial pseudogene, i.e., within the roughly 650 bp region of the ca. 900 bp *Hind *III double digestion product of Figure 5 that contains the bulk of the pseudogene. However, such proximal duplications have not been seen in plant mtDNAs; instead these very large and mostly noncoding genomes rearrange at very high rates, and duplicates are relatively far apart from each other [8,10,11,14,23,33].

A second important line of evidence consistent with a nuclear location of the intact *rps14 *gene of *Carex *is that its reading frame is extended significantly at its 5' end, by at least 32 codons. Such extensions are uncommon in mitochondrial-resident genes, but are typical of mitochondrial genes transferred to the nucleus [2,3], where they commonly serve as cleavable targeting sequences that direct import into the mitochondrion (the *Carex *5' extension is predicted by TargetP v1.1 [34] to be a signal peptide, but not specifically to target to the mitochondrion). It seems particularly unlikely that an essentially adjacent duplication of *rps14 *within the mitochondrial genome would have occurred in such a manner as to create a perfect duplication of the normal-length *rps14 *coding sequence without any trace of *rpl5*, which in virtually all angiosperms is separated by only 1 NT from the 5' end of *rps14 *(see below).

Overall, therefore, these two lines of evidence strongly suggest that the intact *rps14 *gene of *Carex *is not located in the same genome as the *Carex *mitochondrial pseudogene. Because mitochondrial sequences have never been found transferred to plant chloroplast genomes, but are frequently transferred to the nucleus [2,19], and because chloroplast DNA is invariably present in higher copy than mtDNA, a chloroplast location of the intact *Carex rps14 *gene can be ruled out. By default, therefore, this gene is almost certainly present in the nuclear genome.

The second, intact copy of *rps14 *from *Joinvillea *is also not notably more divergent than its RNA-edited – and thus indisputably mitochondrial – pseudogene copy (Figure 3), although in this case neither gene is particular divergent. A similar Southern blot analysis (Figure 5) as for *Carex *also reveals that the mitochondrial pseudogene of *Joinvillea *is present in much higher copy number than the intact copy of *rps14 *(as with *Carex*, the double-digestion enzyme *Hind *III cuts the pseudogene 70 bp from its 5' end but does not cut the intact gene). Two of the double-digests also localize the predominant mitochondrial *rps14 *hybridization signal in *Joinvillea *to a small fragment of about 1 kb, again reducing the possibility of intragenomic *rps14 *duplication to immediately adjacent duplication. Despite considerable effort (see Methods), we did not succeed in obtaining sequence upstream of the normal *rps14 *start codon for the intact *rps14 gene *of *Joinvillea*, and in this sense, therefore, the case for its nuclear location is weaker than for *Carex*. Nonetheless, we think it likely based on the Southern blot results that this gene is in the nucleus. At the same time, we cannot rule out the possibility that the putatively nuclear copy of *rps14 *is in fact located in the mitochondrial genome, but present only on a "sublimon" form of the genome of sufficiently low copy number relative to the principal genome conformer as to be undetectable at the sensitivity level of the Southern blots shown in Figure 5.

### Number of *rps14 *transfers and evolution of transferred *rps14 *genes

Although we have not shown that the putatively nuclear *rps14 *genes of *Carex *and *Joinvillea *are expressed, much less that their products are targeted to the mitochondrion, what seems relatively clear (especially in *Carex*) is that in both plants the mitochondrial genome contains only a single, pseudogene copy of *rps14*. Therefore, the functional copy of *rps14 *almost certainly resides in the nucleus in *Carex *and *Joinvillea*, and since these are the only intact genes isolated via PCR, they are the best candidates for being the functional, transferred copy of *rps14*. That the *Carex *nuclear gene has an intact open reading frame in the face of substantial sequence divergence (Figure 3) strongly supports it being under selection and therefore functional, as does the fact that its estimated ratio of non-synonymous to synonymous substitution (*d*_N_/*d*_S_) is substantially lower (0.28) than 1.0. Nuclear *rps14 *of *Joinvillea *displays relatively little divergence (Figure 3; Additional File 3), and therefore its intactness is not very compelling evidence for it being functional. Strong evidence, however, comes from its *d*_N_/*d*_S _ratio, of 0.16.

Accepting that the intact *rps14 *genes of *Carex *and *Joinvillea *do indeed represent cases of functional transfer to the nucleus, then we can ask whether they and the set of transferred genes in grasses result from a single common transfer, or from two or three independent transfers. Taken together, four lines of evidence, none individually strong, lead us to conclude that these three sets of transferred genes are probably the product of three separate transfer events. First, the three nuclear gene sets do not group together in phylogenetic analysis (Figure 3), as would be expected if they were the product of a single common transfer. At the same time, we must acknowledge that the separation of the three nuclear gene sets is not robust, and that none of the three attaches specifically to its cognate mitochondrial gene clade, as expected under the hypothesis of three separate transfers, specifically in the last common ancestors of the grasses, the Joinvilleaceae, and the Cyperaceae. On the other hand, given the very strong grouping of the entire clade of transferred genes of grasses (including the basal grass *Anomochloa*), and that *Joinvillea *is the closely related sister group of grasses (Figure 2), its failure to group with the grass genes (Figure 3) suggests separate transfers.

Second, and related to this last point, the nuclear *rps14 *of *Joinvillea *is not very divergent in general and is far less divergent than the nuclear genes of grasses (Figure 3). Nuclear substitution rates do not appear to be unusually low in *Joinvillea *[35,36], and therefore this result suggests more recent – and thus independent – transfer of *rps14 *in *Joinvillea *compared to grasses.

Third, and here we have data relevant only to the *Carex *and grass sets of nuclear genes (see Methods), the 5' extension (putative targeting peptide) of the *Carex *nuclear gene is not evidently homologous to those of grasses, which are well conserved among themselves (Figure 4). Possession of different N-terminal extensions has generally been regarded as good evidence for independency of transfer (e.g., [20,31,37]).

Finally, using nine sets of primers designed to *rps14 *and the first exon of *sdh2*, we failed to recover any PCR products from *Carex *and *Joinvillea *indicative of *rps14 *insertion into the *sdh2 *intron under conditions in which all three grasses examined (including the basal grass *Anomochloa*) yielded an *sdh2*-*rps14 *product. This result is consistent with the hypothesis that *rps14 *is in a different location in the *Carex *and *Joinvillea *nuclear genomes than in the grasses and is therefore the product of separate transfers. An important positive control on these negative *sdh2-rps14 *amplifications from *Carex *and *Joinvillea *is that the same *sdh2 *and *rps14 *primers *did *work for each plant in gene-specific contexts. That is, the (3') *rps14 *primers did recover the putatively nuclear *Carex *and *Joinvillea rps14 *genes when used in combination with 5' *rps14 *primers, and the *sdh1*-exon 1 primers did work in concert with primers to *sdh2*-exon 2. Unfortunately, however, we did not sequence the *sdh2 *PCR products from *Carex *and *Joinvillea *to rule out the possibility, albeit remote, that they contain a transferred *rps14 *gene that is, for reasons unclear, refractory to *sdh2*-*rps14*-based PCR isolation (note that the *sdh2 *intron is generally a few times larger than *rps14*).

Although three of the above four lines of evidence are consistent with the *Joinvillea *and grass genes being the product of separate transfers (recall that evidence for *Joinvillea *is lacking for the fourth line of evidence, concerning homology of 5' extension sequences), there is one, ambiguous piece of evidence that, upon one possible interpretation, supports the genes being derived from the same transfer event. This involves the ambiguously aligned 6 NT at positions 89–94 (Additional File 1). According to the "best" alignment, as shown in the figure, mitochondrial *rps14 *genes of *Joinvillea *and grasses contain different deletions in this region, each of 4 NT in length and overlapping by 3 NT. According to this alignment, therefore, the *Joinvillea *and grass genes were pseudogenized independently of one another. Alternatively, by sliding either 5 NT gap by 1 NT, and therefore at the cost of postulating an additional NT substitution in one lineage or the other, one obtains an alignment in which the grasses and *Joinvillea *share a 5 NT deletion. This, then, would constitute evidence that their pseudogenes are the product of a common pseudogenization event. If so, this would also be consistent with the hypothesis that their nuclear genes result from a common transfer event. Overall, then, the case for separate transfer in *Joinvillea *is weaker than for *Carex *(all this relative to grasses). On balance, however, we believe the evidence – in particular based on highly differential degrees of nuclear gene divergence (Figure 3) and failure to isolate a grass-like *sdh2-rps14 *gene under positively controlled circumstances – favors a separate putative transfer in *Joinvillea *as well.

## Discussion

### Multiple transfers of *rps14 *to the nucleus in Poales

We have described three, most likely independent cases of functional transfer of *rps14 *from the mitochondrion to the nucleus during the evolution of the Poales. The transfer in the grass family Poaceae had already been described for the three cereals of greatest agronomic import, *Oryza *(rice), *Zea *(maize), and *Triticum *(wheat) [9,18,20]. Our results indicate that this transfer most likely occurred in the stem lineage leading to all extant grasses (Poaceae), roughly 80 million years ago [38-40]. This conclusion follows from three observations: 1) The previously unexamined basal lineages of grasses (represented by *Anomochloa*, *Streptochaeta*, and *Pharus*) contain a pseudogene copy of *rps14 *in the mitochondrial genome that shares two frameshift mutations with all other grasses. 2) *Anomochloa *contains the same chimeric, *sdh2*/*rps14 *nuclear gene structure as previously described in *Oryza*, *Zea*, and *Triticum*. 3) *Joinvillea*, which is only one node removed from being the sister group to Poaceae (we were unable to obtain material for the obscure sister group to Poaceae, the Ecdeiocoleaceae) appears to share with grasses neither the two mitochondrial *rps14 *frameshifts (but see preceding paragraph) nor the nuclear *sdh2*/*rps14 *gene structure.

The second transfer, apparently (Additional File 1) shared by all three, diverse members of the Cyperaceae examined, is also roughly 80 million years old [39], and possibly older if shared by Juncaceae and even more distantly related Poales (although this possibility cannot be ruled out by our data, there is also no evidence that the transfer occurred prior to the Cyperaceae stem lineage). The third putative transfer, which is the least well supported overall and in terms of being independent (i.e., of the "grass" transfer), is unique to the single examined species of *Joinvillea *(the sole genus in the Joinvilleaceae) and appears to be rather recent as judged by the relatively undiverged nature of the *Joinvillea *nuclear *rps14 *gene (Figure 3). Indeed, based on nuclear divergence levels, this is the most recent case of functional mitochondrial-to-nucleus gene transfer yet described (e.g., compare to [30,31]). A fourth case of *rps14 *functional transfer in Poales is likely represented by *Lachnocaulon*. Southern blot hybridization indicated that *rps14 *is probably missing from *Lachnocaulon *mtDNA [4], however, material of *Lachnocaulon *was not available for this study.

Although the Cyperaceae was included in the Adams et al. [4] survey for mitochondrial gene loss and potential nuclear transfer, its *rps14 *transfer was not inferred because the mitochondrion genome of Cyperaceae contains an essentially full-length pseudogene of *rps14 *(Additional File 1). The *rps14 *transfer in *Joinvillea*, which was not included among the 280 angiosperms surveyed by Adams et al. [4], would have been missed for the same reason. These observations underscore the potential, as discussed by Adams et al. [4], for undiagnosed pseudogenes to lead to underestimates of mitochondrial gene "loss" and potential transfer in such Southern blot surveys. As noted in Background, *rps14 *pseudogenes have also been described in four disparate eudicots [12-17]. Considering all this, and that 27 phylogenetically separate losses of mitochondrial *rps14 were *inferred by Adams et al. [4], it is possible that this gene has actually been transferred to the nucleus many more times than this among the 280 angiosperms examined in that study. On the other hand, as Adams et al. [4] point out, gene loss patterns can also lead to overestimates of the number of gene transfer events, i.e., when phylogenetically separate losses as inferred from blots trace back to a single deeper loss of mitochondrial gene function – and probably nuclear gene transfer – with one or more taxa within the group in question having a largely intact pseudogene scored as present.

As noted in Background, two of the 27 losses of mitochondrial *rps14 *inferred by Adams et al. [4] have been confirmed by complete genome sequencing [8,10], but a third was misdiagnosed, as *Nicotiana *mtDNA actually contains a pseudogene copy comprising most of *rps14*. An additional Southern blot hybridization (K. L. Adams and J. D. Palmer, unpublished) has revealed that *rps14 *is indeed present in mtDNA of the same *Nicotiana *sample examined by Adams et al. [4]. Further inspection of the autoradiogram from which mitochondrial *rps14 *absence was originally and erroneously inferred suggests that this error was caused by poor Southern-blot transfer in the region of the *Nicotiana *lane where the *rps14 *band should have appeared. We emphasize, however, that the overall accuracy of the Southern blot estimates by Adams et al. [4] of mitochondrial gene/presence is very high as assessed by comparison with the definitive results obtained by mitochondrial genome sequencing. Six of the 280 genomes surveyed by Adams et al. [4] have now been sequenced [8,10,11,14,23,33]. These genome sequences confirm all 197 mitochondrial protein gene presences inferred for these six genomes by Adams et al. [4], as well as 41 of 43 gene absences. [Two genes that were detected by Adams et al. [4] using Southern blots, although present in the mitochondrial genome sequences of *Triticum *and *Beta*, were not annotated as such in the GenBank files and were also left out in the publications reporting these genomes. The *Triticum *case [33] involves a nearly intact *rps14 *pseudogene (also see [20]), while the *Beta *case [10] involves a nearly intact *sdh4 *pseudogene.] The second erroneously inferred gene absence was a misdiagnosis by Adams et al. [4] of *rps1 *as absent from mtDNA of *Nicotiana *(and two other members of the Solanaceae: *Capsium *and *Petunia*), when in fact the *Nicotiana *mitochondrial genome sequence shows *rps1 *to be present and intact [11]. This reflects a data-entry error in Figure 2 of Adams et al. [4], as our notebooks show that *rps1 *was correctly scored based on Southern blots as present in all three Solanaceae mtDNAs.

### Prolonged retention, transcription, and editing of *rps14 *pseudogenes

Fully 33 of the 39 diverse genera examined from the three gene-transfer families (Poaceae, Cyperaceae, and Joinvilleaceae) have retained essentially full-length *rps14 *pseudogenes in the mitochondrial genome, with the six exceptions representing but a single case of relatively recent loss of mitochondrial *rps14 *in the grass subfamily Panicoideae (Figure 2). In the case of the Poaceae and Cyperaceae, which represent 32 of the 33 described pseudogenes, this retention is despite the antiquity of the functional transfers to the nucleus and the evident (Additional File 1) pseudogenizations of mitochondrial *rps14*, the latter occurring roughly 80 million years ago in both families. Equally if not more remarkably, all 21 mitochondrial *rps14 *pseudogenes examined (19 in this study, plus *Oryza *[18] and *Triticum *[20]) are transcribed, and 15 of the 21 are subject to RNA editing (Table 1; Additional File 1).

Such long-term, nearly pervasive survival of transcribed (and, usually, RNA-edited) pseudogenes is to our knowledge unparalleled. What could account for this, as well as the presence of virtually intact mitochondrial *rps14 *pseudogenes in a number of eudicots [12-17], including long-lived and pervasively retained pseudogenes in the Solanaceae (H. C. Ong and J. D. Palmer, unpublished data)? First, we could be wrong in calling these pseudogenes. Perhaps *rps14 *is subject to some form of programmed translational recoding (either frameshifting or bypassing; [41]) to produce functional mRNA and protein from these *apparent *pseudogenes. Although programmed recoding is quite rare overall, it has been reported for a number of genes and organisms [41], and should not be entirely dismissed as a possibility here. Sequencing of mitochondrial RPS14 proteins and investigation of potential ribosomal association of mitochondrial *rps14 *transcripts (mRNAs?) could be pursued to test this possibility, unlikely as it may seem (that transferred, putatively functional copies of *rps14 *are present in the nucleus in all examined plant lineages possessing mitochondrial *rps14 *pseudogenes certainly argues against the recoding hypothesis, as does the apparent drift towards noisy, likely unselected patterns of RNA editing; see below).

Second, the low rate of loss and decay of mitochondrial *rps14 *pseudogenes may, at least in part, simply reflect generally low mutation rates in plant mitochondrial genomes. It is well known that, with rare exception [42,43], synonymous substitution rates are extremely low in plant mitochondrial genomes [28-30]. Comparable data on neutral rates of both short indels (e.g., those leading to the short frameshift mutations highlighted in Additional File 1) and long indels (those leading to gene loss) are lacking for plant mtDNAs. However, if the positive correlation observed in other genomes (e.g,. [44]) between rates of substitutions and rates of indels holds true for plant mitochondrial genomes, then this may explain part of the *rps14 *pseudogene paradox. Indeed, the alignments shown in Additional File 1 (also see Figure 2) suggest that rates of both substitutions and short indels are elevated in the Cyperaceae. However, whether large deletions leading to gene loss are also elevated in Cyperaceae (and in general correlate with rates of substitutions and small indels) remains to be seen. A very different picture is seen for the only other gene transfer case (involving *cox2 *in legumes; [45]) for which extensive sampling of mitochondrial gene/presence and gene sequence has been carried out within the transfer clade. There, a largely all-or-nothing pattern is seen; among 21 legume genera belonging to the gene transfer clade, only a single mitochondrial pseudogene was found, compared to three independent cases of *cox2 *gene loss and several lineages in which an intact and probably functional mitochondrial *cox2 *gene was found [45].

Third, independent of its protein-coding function, there may be other functional elements within or in close proximity to *rps14 *that make it difficult or impossible to delete (much more so than *cox2*) even when it is a pseudogene. Most angiosperm mitochondrial genes (including *cox2*) occur as a single genic island in a large sea of intergenic spacer DNA [8,10,11,14,23,33]. However, *rps14 *is exceptional in that it is cotranscribed with [13,15,18,22,24] and separated by only a single NT from *rpl5 *in virtually all angiosperms (see references in Background; our unpublished results; the only known exceptions are *Oenothera *and *Vicia *[21,25]). By being so short (barely 300 NT) and so tightly linked to *rpl5*, *rps14 *may be relatively buffered compared to most plant mitochondrial genes against large deletions that would lead to gene loss. Another possibility is that sequences within *rps14 *or immediately downstream of it (e.g., a transcription terminator) are under selection to maintain proper expression of *rpl5*. While these arguments may hold water for Cyperaceae, for which *rpl5 *is intact and immediately upstream of *rps14 *(our unpublished data), they are problematic for grasses. This is because *rpl5 *has been functionally transferred to the nucleus in most grasses ([20]; our unpublished data), and despite its multiple, within-grass transfers being more recent than the grass-wide *rps14 *transfer and pseudogenization, there have been fully five independent losses of mitochondrial *rpl5 *among the same 35 grasses examined in this study (our unpublished data), while some (but not all) other grasses contain *rpl5 *pseudogenes in the mitochondrion. The pervasive and prolonged (ca. 80 million year) retention of mitochondrial *rps14 *pseudogenes in grasses and Cyperaceae also contrasts with the numerous (26, discounting the *Nicotiana *error) phylogenetically separate losses of mitochondrial *rps14 *across the broad sweep of angiosperm phylogeny [4].

Overall, then we are left with a suspicion that *rps14 *may be unusually recalcitrant – even as a pseudogene – to elimination from mitochondrial genomes of Poaceae and Cyperaceae. The possibility that some portion of the *rps14 *sequence may play some entirely unrelated role that renders the pseudogene's deletion problematic must be considered, as must the possibility that it's not actually a pseudogene thanks to some form of programmed translational recoding. On the other hand, *rps14 *and *rpl5 *may simply lie near different ends of the expected frequency distribution of pseudogene deletion in such slowly evolving genomes as those of plant mitochondria (note also that despite the five *rpl5 *losses, many other grasses retain virtually intact *rpl5 *pseudogenes; our unpublished data).

The continued, presumably meaningless transcription of mitochondrial *rps14 *(and *rpl5 *too; unpublished data) in many grasses in which both mitochondrial genes are, apparently, defunct, is not unexpected given that plant mitochondrial promoters are very short [46,47] and that rates of nucleotide substitutions and short indels are very low. These pseudo-transcripts continue to be edited at low levels comparable to those of intact and putatively functional mitochondrial *rps14 *genes (Table 1). However, two observations suggest that this represents noisy/leaky editing that is not under selection. First, fully half of the edits are U→C edits, which are extremely rare in angiosperm mitochondrial genes (reviewed in [48]). Indeed, all of the 357–491 edits detected in the four angiosperms in which comprehensive analyses of RNA editing have been carried out are C→U edits ([19,23,49]; Mower and Palmer, submitted). Second, many of the *rps14 *pseudogene edits (of both types) reduce the conservation of RPS14 proteins (see Additional File 2), whereas 94% of nonsynonymous edits increase protein conservation across species [50-53]. Note that transcribed and edited pseudogenes have also been described for several other mitochondrial genes in a diversity of angiosperms ([12,13,15,54,55]; Mower and Palmer, submitted).

The peculiar evolutionary dynamics of plant mitochondrial genes and their expression leads to a conundrum with respect to using gene sequence and expression data to predict whether a given gene is likely to be functional or not. Because transcribed and RNA-edited pseudogenes are relatively common in plant mtDNA, and because rates of gene decay/pseudogenization are relatively low (Additional File 1), one can predict that an appreciable fraction of *intact *genes that are transcribed and RNA-edited are nonetheless not functional. Because many plant mitochondrial pseudogenes show evidence of relaxed constraints on editing ([15,20,53,56]; Mower and Palmer, submitted), this may be a second important source of clues (in addition to typical pseudogenization mutations in the gene itself) that a gene is non-functional. Indeed, the loss of editing at evolutionarily conserved positions has led to the suggestion that the intact *rps14 *gene of *Brassica *[23] and *rps1 *gene of *Oenothera *[57] are in fact cryptic pseudogenes. This issue is particularly relevant to those two classes of plant mitochondrial protein genes (their 14 ribosomal protein genes and two succinate dehydrogenase genes) that are very frequently lost from the mitochondrial genome and functionally transferred to the nucleus during angiosperm evolution [4]. Many of the intact mitochondrial copies of these genes may turn out, upon further (especially cDNA) analysis, to instead be cryptic pseudogenes.

### Indels in *rps14 *pseudogenes

Three aspects of the pattern and apparent frequency of indels in the mitochondrial *rps14 *pseudogenes deserve comment. The first was noted above, that the Cyperaceae pseudogenes have on average sustained more indels (mostly frameshifts) than the equal-aged Poaceae pseudogenes, and that this may reflect mechanistically-related, correlated increases in rates of nucleotide substitutions and small indels in the Cyperaceae (Additional File 1). Second, there may be an elevated rate of indels in the ~20 million year lineage leading to *Eragrostis*. This lineage has accumulated four frameshifts, compared to no indels in about half of the grass pseudogenes (excluding the two deletions that occurred in the common ancestor of all grasses) and only a single indel in most other grasses (Additional File 1). Unlike Cyperaceae, *Eragrostis *does not show an elevated substitution rate, and if this pattern holds with much more data (and it may well not; *Eragrostis *could simply be a stochastic outlier), then this could be an apparently rare case of uncoupled rates of nucleotide substitutions and short indels.

Finally, there are two sets of apparently homoplasiously derived indels (Additional File 1). None of these involve short direct repeats, where replication slippage can readily produce such patterns of homoplasy. Instead, because the same pattern extends across about 10 markers in the adjacent *rpl5 *gene, we believe that the second set (positions 286–289) of indel homoplasies is the result of horizontal gene transfer from *Festuca *to *Secale *and *Danthonia*. Horizontal transfer could also explain the second set of homoplasies (positions 198–202), but there is far less evidence for transfer in this case (this homoplasious deletion being the only evidence), and parallel deletion is a good alternative explanation. These and many additional cases of more or less well supported horizontal transfer of the *rpl5*/*rps14 *locus will be the subject of a forthcoming paper (H.C. Ong, D.W. Rice, S.M. Chang, and J.D. Palmer, in preparation).

## Conclusion

Our findings show that the mitochondrial *rps14 *gene has, most likely, been functionally transferred to the nucleus at least three times during the evolution of the Poales. We extend the one previously recognized transfer from a limited number of grasses to the full breadth of grasses and date its occurrence to roughly 80 million years ago. We describe two new cases of *rps14 *transfer: a comparably old transfer in the Cyperaceae and a much younger, putative transfer in the Joinvilleaceae. Surprisingly, despite the great age of the Poaceae and Cyperaceae transfers (and of the apparent loss of functionality of the original mitochondrial *rps14 *genes), nearly intact and expressed pseudogenes of *rps14 *have persisted in the mitochondrial genomes of most lineages of the two families. To our knowledge, such long-term, nearly pervasive survival of expressed pseudogenes is unparalleled in any other gene or genome. Why this is so is not entirely clear. It probably reflects a combination of factors, including the simple nature of plant mitochondrial expression sequences and the short length of *rps14 *(making them small targets for disabling mutations), low rates of nucleotide substitutions and indels in plant mtDNAs, and (especially in Cyperaceae) the presence of an intact, functional *rpl5 *gene immediately upstream of *rps14*. Such survival also raises the possibility that, at least in the Poales, *rps14 *may harbour some functionally important sequence element that is unrelated to its ribosomal-protein-encoding function. Overall, these findings indicate that intracellular gene transfer may occur even more frequently in angiosperms than already recognized and that pseudogenes in plant mitochondrial genomes can be surprisingly resistant to forces that lead to gene loss and inactivation.

## Methods

### Slot and Southern blot hybridization

Slot blot filters were made by denaturing 300 ng of total plant DNA with 0.4 M NaOH and loading the DNA onto a nylon membrane (Millipore) clamped in a Minifold II slot apparatus (Schleicher & Schuell). Southern blot filters were made by digesting total DNAs with restriction enzymes for 2 hours, separating fragments on 0.8% agarose gels, and transferring fragments from gels to nylon membranes using the method of Dowling et al. [58]. Probes made with 75 ng of ^32^P-labeled gene-specific PCR products were hybridized to membranes at 60°C overnight in 5 × SSC, 50 mM Tris (pH 8.0), 0.1% SDS, 10 mM EDTA, 2 × Denhardt's solution and 5% dextran sulfate (Amersham Pharmacia). Membranes were washed twice with 2 × SSC at room temperature and twice at 60°C with 2 × SSC, 0.5% SDS.

### Gene isolation and characterization

Genomic DNAs were obtained from other labs or isolated from leaves using the method of Doyle and Doyle [59]. PCR amplification of *rps14*, from either genomic or cDNA templates, was conducted using primers rps14F1(ATGWYGGAGAAGCRAAATAKA), rpl5F1 (ATGYTTCCRCTCHATWTTCAT), rps14R1(TACCAAGACGATTTCTTTATG), rps14R2 (GGACTAGCTGCAGAGCTAACC), nuS14F6B (CRCAAACGTAGAHTKCTYGC), nuS14F7 (GCKKGAYCRCAAACGTAGA), and nuS14R3 (CTACCAHGAYGCYTTCTTWAC). To amplify the nuclear *sdh2*-*rps14 *locus, primer sdh2F (CTCCACATCCTGCCCGTCCT) was used together with the reverse *rps14 *nuclear primer. To isolate the *Carex *sequence upstream of *rps14*, we used the Vectorette II kit (Sigma-Genosys) with primers CarINT (GCTAGCGTACGGAAAACAATA) and nuS14R3; genomic *Carex *DNA was digested with *BamH *I and ligated to the *BamH *I Vectorette end. All 5 Vectorette ends (4 sticky and 1 blunt) were used in our attempt to isolate *Jonvillea*'s 5' extension but all attempts failed. Also unsuccessful were efforts to obtain farther upstream sequences for *Carex *by 5' RACE, as were 5' RACE efforts for *Joinvillea*. Thermocycling parameters were 35 cycles of 94°C, 3 minutes; 54°C, 30 seconds; and 72°C, 40 seconds. PCR products were purified using ExoSAP-IT (United States Biochemical) and sequenced directly using an ABI 3730 (Applied Biosystems) at the Indiana Molecular Biology Institute. Sequence alignment used Se-Al v2.0 a11 (Oxford, UK).

For RT-PCR, RNA was isolated using the RNeasy Plant Mini Kit (Qiagen) and treated twice with DNase I (TaKaRa). A total of 1 μg RNA was reverse transcribed using Moloney-murine-leukaemia-virus reverse transcriptase (New England Biolabs) with random hexamers (Invitrogen). The cDNAs were then PCR-amplified, cloned with TOPO TA Cloning Kit (Invitrogen), and sequenced. The following controls were conducted to ensure that the persumptive cDNA products were indeed produced from RNA templates: 1) Total RNAs were treated twice with DNaseI and re-precipitated after each treatment, and 2) PCR amplification alone (using random primers and without prior reverse transcription) was conducted with each of the DNAse-treated RNAs as template and yielded no amplified products. Therefore these appear to be bona fide cDNAs rather than PCR products from genomic DNA contamination of the RNA preparations. Note that all plants for which cDNA sequences were obtained were also sequenced for the corresponding genomic sequence, i.e., the inferred RNA edits are real and not the result of natural polymorphisms stemming from use of different plants for generation of cDNA and genomic sequences. Finally, all edits were inferred from clean sequence reads, generated from both strands of a clone, and most were also validated by obtaining the same sequence from multiple independent cDNA clones (Table 1).

All sequences generated in this study have been submitted under accession numbers [Genbank:DQ380465–DQ380508]. Sequences we did not generate but used are: *Arabidopsis *[Genbank:X67736], *Hordeum *[GenBank:BE438633, BE438684. CA009776], *Oryza *[GenBank:AB017429, AB076666, BI811279, BU673503, CA766255, CB966695, CB966711], *Triticum *[GenBank:AJ539160, BE593854, BJ249329, CA648962, CD939079, CD939080], *Sorghum *[GenBank:AW922306, BE593854, CF487648] and *Zea *[GenBank:AJ012374, CF244985].

### Phylogenetic analyses

Maximum likelihood analyses were performed using PAUP v. 4.0b10 (Swofford) [60]. Trees were constructed using the HKY85 substitution model, estimated transition to transversion ratio, approximated gamma distribution and four rate categories. All trees were swapped by random stepwise addition with TBR branch swapping. Bootstrap values were obtained from 100 replicates using the same HKY85 substitution model.

## Authors' contributions

HCO carried out all of the studies reported in this paper, while JDP conceived the study and provided frequent input into experimental design and interpretation. The two authors jointly wrote the manuscript. Both authors read and approved the final manuscript.

## Supplementary Material

Additional File 1 Sequence alignment of selected mitochondrial *rps14 *genes. Sequences are aligned relative to the inferred ancestral monocot *rps14 *sequence with identical nucleotides indicated by dots, gaps by dashes, and missing data by "~". All frameshift indels are marked in colors by family: Poaceae (red), Joinvilleaceae (orange), and Cyperaceae (blue). *rps14 *cDNAs were sequenced from shaded genera. Sites of RNA editing are shown as gray boxes (C→U edits) or black boxes with white lettering (U→C edits). Click here for file

Additional File 3 DNA sequences of all Poales mitochondrial and nuclear *rps14 *sequences used in this study. Mitochondrial sequences shown here are the same as in Additional File 1, also included are all putatively nuclear *rps14 *sequences from Poales (see Figure 3). Click here for file

Additional File 2 RPS14 amino acid sequence changes as a result of RNA editing. The table shows the specifics of RNA editing for 15 of the 21 taxa shown in Table 1. cDNA sequences for the other six taxa (*Luzula*, *Oryza*, *Glyceria*, *Poa*, *Bromus*, *Phragmites*; the *Oryza *cDNA is from Kubo et al. [18] and the *Triticum *cDNA is from Sandoval et al. [20]) are not shown because they are not RNA-edited. cDNA translations disregard indels, i.e., they assume an intact open reading frame. Click here for file
