# Supplementary figures and images for: Pervasive survival of expressed mitochondrial rps14 pseudogenes in grasses and their relatives for 80 million years following three functional transfers to the nucleus
Source: BMC Evol Biol. 2006 Jul 14;6:55. doi: 10.1186/1471-2148-6-55 (PMC1543663; doi:10.1186/1471-2148-6-55)

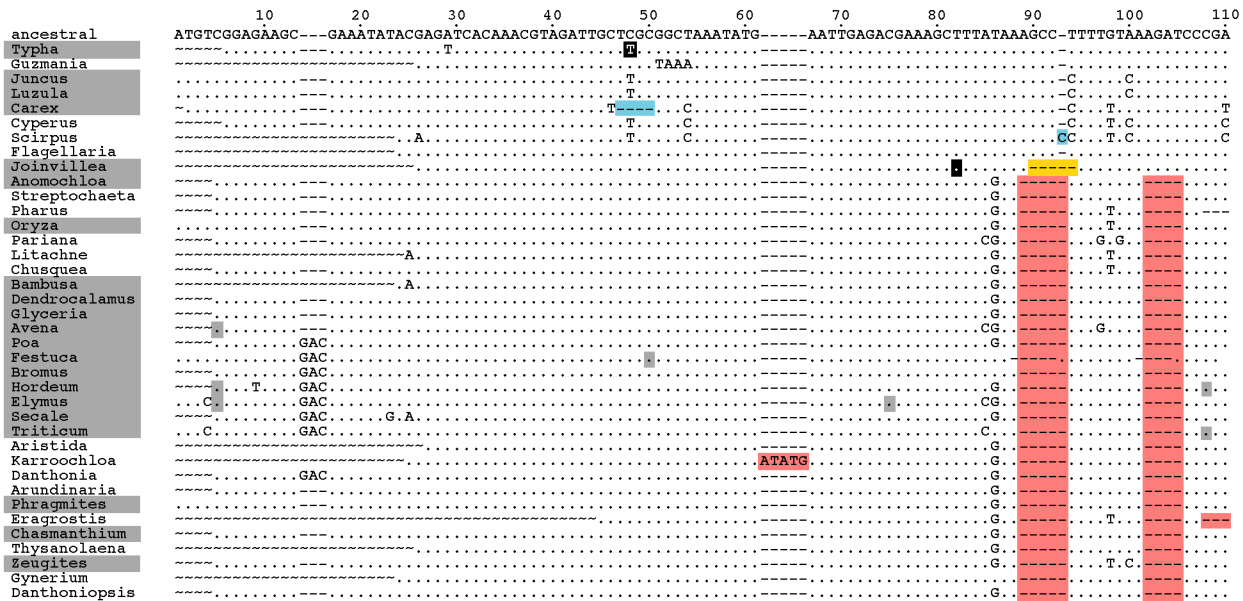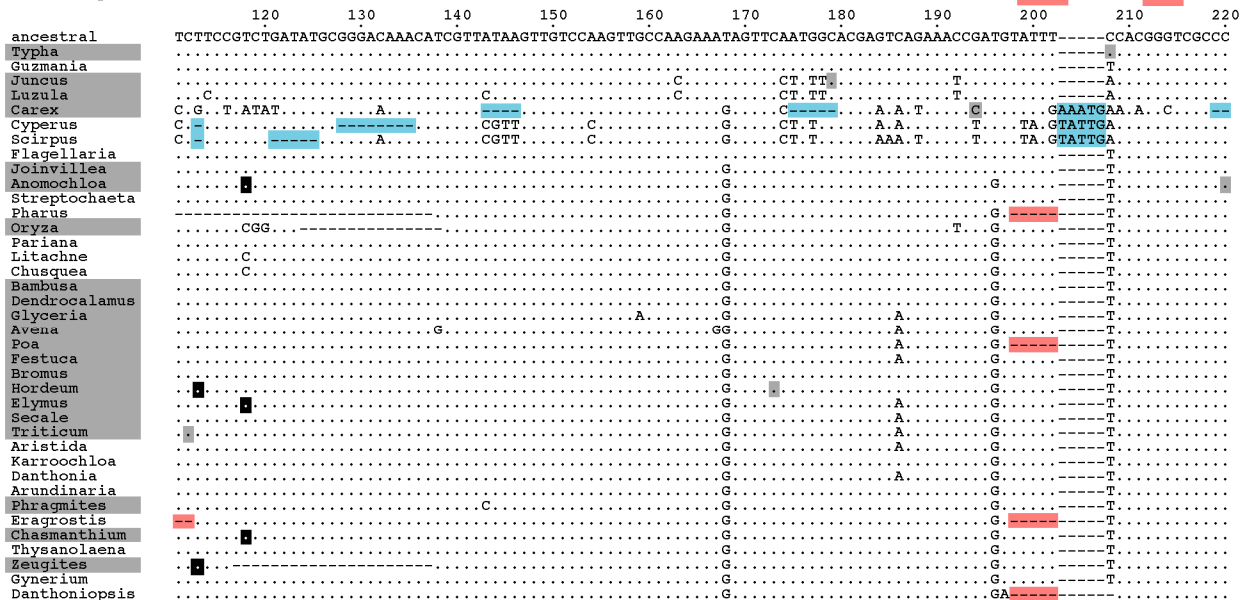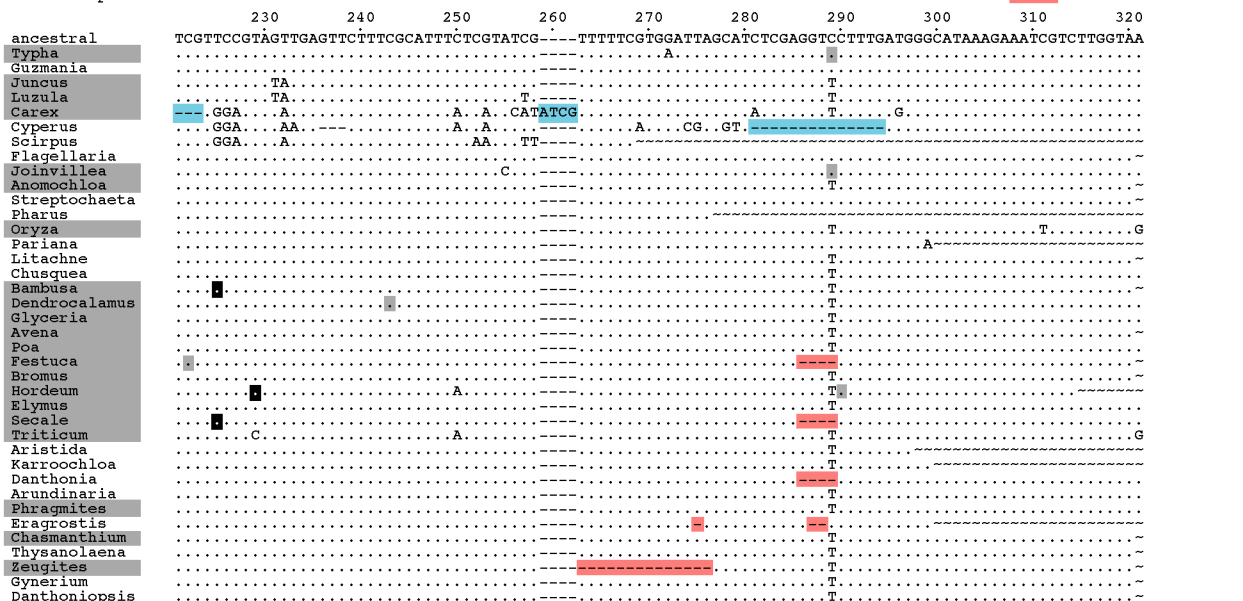

Supplement: Additional File 1 — Sequence alignment of selected mitochondrial rps14 genes. Sequences are aligned relative to the inferred ancestral monocot rps14 sequence with identical nucleotides indicated by dots, gaps by dashes, and missing data by "~". All frameshift indels are marked in colors by family: Poaceae (red), Joinvilleaceae (orange), and Cyperaceae (blue). rps14 cDNAs were sequenced from shaded genera. Sites of RNA editing are shown as gray boxes (C→U edits) or black boxes with white lettering (U→C edits). [file 1471-2148-6-55-S1.pdf]
