# Supplementary material for: Pervasive survival of expressed mitochondrial rps14 pseudogenes in grasses and their relatives for 80 million years following three functional transfers to the nucleus
Source: BMC Evol Biol. 2006 Jul 14;6:55. doi: 10.1186/1471-2148-6-55 (PMC1543663; doi:10.1186/1471-2148-6-55)
Supplement: Additional File 3 — DNA sequences of all Poales mitochondrial and nuclear rps14 sequences used in this study. Mitochondrial sequences shown here are the same as in Additional File 1, also included are all putatively nuclear rps14 sequences from Poales (see Figure 3). [file 1471-2148-6-55-S3.pdf]

```
[                        10        20        30        40        50        60        70        80        90       100       110]
[                         .         .         .         .         .         .         .         .         .         .         .]

ancestral        ATGTCGGAGAAGC---GAAATATACGAGATCACAAACGTAGATTGCTCGCGGCTAAATATG-----AATTGAGACGAAAGCTTTATAAAGCC-TTTTGTAAAGATCCCGA   [101]
Typha            ~~~~~........---............T..................T.............-----..........................-.................   [96]
Guzmania         ~~~~~~~~~~~~~~~~~~~~~~~~~.........................TAAA.......-----..........................-.................   [79]
Juncus           .............---...............................T.............-----..........................-C.....C..........   [101]
Luzula           .............---...............................T.............-----..........................-C.....C..........   [101]
Carex            ~............---.............................-.---...C.......-----..........................-C...T...........T   [96]
Cyperus          ~~~~~........---...............................T.....C.......-----..........................-C...T.C.........C   [96]
Scirpus          ~~~~~~~~~~~~~~~~~~~~~~~..A.....................T.....C.......-----..........................CC...T.C.........C   [82]
Flagellaria      ~~~~~~~~~~~~~~~~~~~~~~~......................................-----..........................-.................   [81]
Joinvillea       ~~~~~~~~~~~~~~~~~~~~~~~~~....................................-----.......................-----................   [75]
Anomochloa       ~~~~.........---.............................................-----...................G..-----........----.....   [89]
Streptochaeta    ~~~~.........---.............................................-----...................G..-----........----.....   [89]
Pharus           ~~~~.........---.............................................-----...................G..-----....T...----..---   [86]
Oryza            .............---.............................................-----...................G..-----....T...----.....   [93]
Pariana          ~~~~.........---.............................................-----..................CG..-----...G.G..----.....   [89]
Litachne         ~~~~~~~~~~~~~~~~~~~~~~~~A....................................-----...................G..-----....T...----.....   [72]
Chusquea         ~~~~.........---.............................................-----...................G..-----....T...----.....   [89]
Bambusa          ~~~~~~~~~~~~~~~~~~~~~~~.A....................................-----...................G..-----........----.....   [73]
Dendrocalamus    ~~~~.........---.............................................-----...................G..-----........----.....   [89]
Glyceria         ~~~~.........---.............................................-----...................G..-----........----.....   [89]
Avena            ~~~~.........---.............................................-----..................CG..-----...G....----.....   [89]
Poa              ~~~~.........GAC.............................................-----...................G..-----........----.....   [92]
Festuca          .............GAC.............................................-----...................G..-----........----.....   [96]
Bromus           ~~~~.........GAC.............................................-----...................G..-----........----.....   [92]
Hordeum          ~~~~....T....GAC.............................................-----...................G..-----........----.....   [92]
Elymus           ...C.........GAC.............................................-----..................CG..-----........----.....   [96]
Secale           ~~~~.........GAC......G.A....................................-----...................G..-----........----.....   [92]
Triticum         ...C.........GAC.............................................-----..................C...-----........----.....   [96]
Aristida         ~~~~~~~~~~~~~~~~~~~~~~~~~~...................................-----...................G..-----........----.....   [70]
Karroochloa      ~~~~~~~~~~~~~~~~~~~~~~~~.....................................ATATG...................G..-----........----.....   [77]
Danthonia        ~~~~.........GAC.............................................-----...................G..-----........----.....   [92]
Arundinaria      ~~~~.........---.............................................-----...................G..-----........----.....   [89]
Phragmites       .............---.............................................-----...................G..-----........----.....   [93]
Eragrostis       ~~~~~~~~~~~~~~~~~~~~~~~~~~~~~~~~~~~~~~~~~~~~.................-----...................G..-----....T...----..---   [49]
Chasmanthium     ~~~~.........---.............................................-----...................G..-----........----.....   [89]
Thysanolaena     ~~~~~~~~~~~~~~~~~~~~~~~~~....................................-----...................G..-----........----.....   [71]
Zeugites         ~~~~.........---.............................................-----...................G..-----....T.C.----.....   [89]
Gynerium         ~~~~~~~~~~~~~~~~~~~~~~~......................................-----......................-----........----.....   [73]
Danthoniopsis    ~~~~.........---.............................................-----...................G..-----........----.....   [89]
Anomochloa_N     G....A......A---....CT.GTTG....G.........GA....T..A..........-----.GC.....AA......C.....GT..-A.G....G......T..   [101]
Pariana_N        ~~~~~~~~~~~~~~~~~~~~~~~~~~~~~..G...............T..A..A.......-----.GC.....G.............G..T-G.C....GG..C..T..   [75]
Oryza_N          G.A.........A---....CT.G.TG....................T..A..A.......-----..C...A.G.............G..T-G......GG..C..T..   [101]
```

```
Dendrocalamus_N    G.A..A......A---....CT.G.TG....................T..A..A.......-----.GC.....G.............G...-G.C....GG..C..T..   [101]
Bambusa_N          G.A..AA.....A---.....T.G..G....................T.....A.......-----.GC.....G..C..........G...-G......GG..C..T..   [101]
Poa_N              ..A..A......A---....CT.G..G....................G..A..A.......-----.GC.....G....A........GT.T-G.C....GG..C..T..   [101]
Hordeum_N          G.C..A......A---....C..G..T....................T..A..A.......-----.GC.....G.............GT.T-G.C....GG..C..T..   [101]
Triticum_N         G.C..A......A---....CT.G..G...............A.T..T..A..A.......-----.GC.....G.............G..T-G.C....GG..C..T..   [101]
Pennisetum_N       ~~~~~~~~~~~~~~~~~~~~~~.G..G....G...............T..A.AA.......-----.GC.A...G.......G.....G..T-G.C....GG..C..T..   [82]
Sorghum_N          G....A......A---....CC.G..G..C.................T..A.AA.......-----.GC....GG.......G.....G..T-G.C....GG..C..T..   [101]
Zea_N              G....A......A---....CC.G..G..C....................A.AA..G.T..-----.GC.....G.......G.....G..T-G.C....GG..C..T..   [101]
Joinvillea_N       ~~~~~~~~~~~~~~~~~~~~~~~~~~~~~~~~~~~~~~~~~~~~~~~~~~~~~.C......-----..........G..C............-......C..........   [51]
Carex_N            G..G.A..C....---........G....CT................T..A..C.......-----.G..............G..C......-C.....C........AC   [101]

[                           120       130       140       150       160       170       180       190       200       210       220]
[                           .         .         .         .         .         .         .         .         .         .         .]

ancestral          TCTTCCGTCTGATATGCGGGACAAACATCGTTATAAGTTGTCCAAGTTGCCAAGAAATAGTTCAATGGCACGAGTCAGAAACCGATGTATTT-----TCACGGGTCGCCC   [206]
Typha              ............................................................................................-----C............   [201]
Guzmania           ............................................................................................-----.............   [184]
Juncus             ....................................................C..........CT.TT.............T..........-----A............   [206]
Luzula             ...C............................C...................C..........CT.TT.............T..........-----A............   [206]
Carex              C.G..T.ATAT..........A..........----.....................G.....C-----....A.A.T.....C.......GAAATGAA.A..C......--   [195]
Cyperus            C.-..............--------.......CGTT.......C.............G.....CT.T......A.A.......T....TA.GTATTGA............   [197]
Scirpus            C.-.......-----......A..........CGTT.......C.............G.....CT.T......AAA.T.....T....TA.GTATTGA............   [186]
Flagellaria        ............................................................................................-----.............   [186]
Joinvillea         .........................................................G..................................-----.............   [180]
Anomochloa         .........................................................G...........................G......-----.............   [194]
Streptochaeta      .........................................................G..................................-----.............   [194]
Pharus             ---------------------------..............................G...........................G.----------.............   [159]
Oryza              .......CGG...---------------.............................G.......................T...G......-----.............   [183]
Pariana            .........................................................G...........................G......-----.............   [194]
Litachne           .......C.................................................G...........................G......-----.............   [177]
Chusquea           .......C.................................................G...........................G......-----.............   [194]
Bambusa            .........................................................G...........................G......-----.............   [178]
Dendrocalamus      .........................................................G...........................G......-----.............   [194]
Glyceria           ................................................A........G.................A.........G......-----.............   [194]
Avena              ...........................G............................GG.................A.........G......-----.............   [194]
Poa                .........................................................G.................A.........G.----------.............   [192]
Festuca            .........................................................G.................A.........G......-----.............   [201]
Bromus             .........................................................G...........................G......-----.............   [197]
Hordeum            .........................................................G...........................G......-----.............   [197]
Elymus             .........................................................G.................A.........G......-----.............   [201]
Secale             .........................................................G.................A.........G......-----.............   [197]
Triticum           .........................................................G.................A.........G......-----.............   [201]
Aristida           .........................................................G.................A.........G......-----.............   [175]
Karroochloa        .........................................................G...........................G......-----.............   [182]
Danthonia          .........................................................G.................A.........G......-----.............   [197]
Arundinaria        .........................................................G...........................G......-----.............   [194]
```

```
Phragmites          ................................C........................G...........................G......-----.............   [198]
Eragrostis          --.......................................................G...........................G-----------.............   [147]
Chasmanthium        .........................................................G...........................G......-----.............   [194]
Thysanolaena        .........................................................G...........................G......-----.............   [176]
Zeugites            ......---------------------..............................G...........................G......-----.............   [173]
Gynerium            .........................................................G...........................G......-----.............   [178]
Danthoniopsis       .........................................................G...........................GA-----------............   [188]
Anomochloa_N        ......CATG............C.GTTC.........C.............G...............A....GC.T...........C....-----...........T.   [206]
Pariana_N           ......A..A..G........T..GTT...C...............C.......G........C...A....TC.T.C......C.......-----....A..C...T.   [180]
Oryza_N             ......AG.A.......A....C.GTT...C......C......................C..C...A....CC.T........C.....C.-----.......C...T.   [206]
Dendrocalamus_N     ......A..A...........T..GTT...C......C........C................C...A....TC.T.C......C.......-----....C..C..AT.   [206]
Bambusa_N           ......A..A........A..T..GTT...C......C........C................C...A....TC.T.C......C.......-----....T..C.....   [206]
Poa_N               ...A..A..A...........T..GTT...CG............G.C..............AAC...A....TC.T..G..T..C.......-----.......C..AT.   [206]
Hordeum_N           ......A..A...........T..GTT...C...............C..............AAC...A....TC.T........C.......-----.......C...T.   [206]
Triticum_N          A.....A..A...........T..GTT...C...............C..............AAC...A....TC.T........C.......-----.......C...T.   [206]
Pennisetum_N        ......A.TG...........A..GTTC..C...........A........C........C..C...A....CC.T........C..C..A.-----.T..A..C...T.   [187]
Sorghum_N           ......A.TG...........G..GTTC..C.............................C..C...A....CC.T........C..C....-----....A..C..GT.   [206]
Zea_N               ......A.TG...........G..GTTC..C.....A.......................C..C...A....CC.T........C..C....-----.......A..GT.   [206]
Joinvillea_N        .........................................................G..................................-----.............   [156]
Carex_N             ......A......T................AG.....C........C.....C....C...G.CT.TT....G.....G.....G.....A.-----ATT.......G..   [206]

[                          230       240       250       260       270       280       290       300       310       320]
[                            .         .         .         .         .         .         .         .         .         . ]

ancestral           TCGTTCCGTAGTTGAGTTCTTTCGCATTTCTCGTATCG----TTTTTCGTGGATTAGCATCTCGAGGTCCTTTGATGGGCATAAAGAAATCGTCTTGGTAA   [303]
Typha               ......................................----.........A.................................................   [298]
Guzmania            ......................................----...........................................................   [281]
Juncus              ..........TA..........................----..........................T................................   [303]
Luzula              ..........TA........................T.----..........................T................................   [303]
Carex               ---.GGA....A.................A..A..CATATCG..................A.......T......G.........................   [293]
Cyperus             ....GGA....AA..---...........A..A.....----......A....CG..GT.--------------...........................   [277]
Scirpus             ....GGA....A...................AA...TT----......~~~~~~~~~~~~~~~~~~~~~~~~~~~~~~~~~~~~~~~~~~~~~~~~~~~~   [230]
Flagellaria         ......................................----..........................................................~   [282]
Joinvillea          ..................................C...----...........................................................   [277]
Anomochloa          ......................................----..........................T...............................~   [290]
Streptochaeta       ......................................----..........................................................~   [290]
Pharus              ......................................----............. ~~~~~~~~~~~~~~~~~~~~~~~~~~~~~~~~~~~~~~~~~~~~~   [211]
Oryza               ......................................----..........................T.....................T.........G   [280]
Pariana             ......................................----....................................A~~~~~~~~~~~~~~~~~~~~~~   [269]
Litachne            ......................................----..........................T...............................~   [273]
Chusquea            ......................................----..........................T................................   [291]
Bambusa             ......................................----..........................T...............................~   [274]
Dendrocalamus       ......................................----..........................T................................   [291]
Glyceria            ......................................----..........................T................................   [291]
Avena               ......................................----..........................T...............................~   [290]
Poa                 ......................................----..........................T................................   [289]
```

```
Festuca            ......................................----.......................----...............................~   [293]
Bromus             ......................................----..........................T...............................~   [293]
Hordeum            .............................A........----..........................T.........................~~~~~~~   [287]
Elymus             ......................................----..........................T................................   [298]
Secale             ......................................----.......................----................................   [290]
Triticum           ........C....................A........----..........................T...............................G   [298]
Aristida           ......................................----..........................T........~~~~~~~~~~~~~~~~~~~~~~~~   [248]
Karroochloa        ......................................----..........................T..........~~~~~~~~~~~~~~~~~~~~~~   [257]
Danthonia          ......................................----.......................----................................   [290]
Arundinaria        ......................................----..........................T................................   [291]
Phragmites         ......................................----..........................T................................   [295]
Eragrostis         ......................................----............-...........--T..........~~~~~~~~~~~~~~~~~~~~~~   [219]
Chasmanthium       ......................................----..........................T...............................~   [290]
Thysanolaena       ......................................----..........................T...............................~   [272]
Zeugites           ......................................------------------............T...............................~   [255]
Gynerium           ......................................----..........................T...............................~   [274]
Danthoniopsis      ......................................----..........................T...............................~   [284]
Anomochloa_N       C..CGGT..GTACA..AAA..C.....G..C.....T.----....C...TC.C.GC..AA.AA....GAA....C....G.....~~~~~~~~~~~~~~~   [288]
Pariana_N          ....GGT..CTACA.AAAA..C.....G........T.----.G..C..CTC...G..TAACAAG..CGAAG..T....TG........G.A........G   [277]
Oryza_N            G..CG.T..TTACA..AAA..C.....G........T.----.G..C..GTC...G..TAA.AAG...GAA...T....TG.T......G..........G   [303]
Dendrocalamus_N    ....G.T..CTACA..AAA..C.....G........T.----.G..C..CTC...G...AA.AAG...GAAC...C....G........GAT.T~~~~~~~   [296]
Bambusa_N          ..A.G.T..CTACA..AAA..C.....G........T.----.G..C..CAC..~~~~~~~~~~~~~~~~~~~~~~~~~~~~~~~~~~~~~~~~~~~~~~~   [256]
Poa_N              ....GGT..CTACA..AAA..C.....G..A.......----.G..C..CAC..GCTA..AAGA~~~~~~~~~~~~~~~~~~~~~~~~~~~~~~~~~~~~~   [266]
Hordeum_N          ....GGT..CTACC.AAAA..C.....G..........----.G..C...ACC..G...AA.AAG...GAAC.......TG.T......G.A........G   [303]
Triticum_N         ....GGT..CTACC..AAA..C.....G........T.----.G..C..CACC..G...AA.AAG...GAAC.......TG.T......G..........G   [303]
Pennisetum_N       ....G.T..CTACA..AAA..C.....G..C.....T.----.G..C..CAC...G...AACAAG...GAAC...C...GG.......~~~~~~~~~~~~~   [271]
Sorghum_N          ...CG.T..GTACA..AAA..C.....G..C.....T.----.G..C..CAC...G...AACAAG...GAAC...C....G........G.A..A.....G   [303]
Zea_N              ....G.T..CTACA..AAA..C.....G........T.----.G..C..GAC...G...AACAAG...GAAC.................G.A..G.....G   [303]
Joinvillea_N       ...........A..........................~~~~~~~~~~~~~~~~~~~~~~~~~~~~~~~~~~~~~~~~~~~~~~~~~~~~~~~~~~~~~~~   [194]
Carex_N            ...C..T..GTACAG.C.G.......C.........T.----....C...ACGC........GA....T.C........TG........G.A........G   [303]
```
