# Supplementary material for: Pervasive survival of expressed mitochondrial rps14 pseudogenes in grasses and their relatives for 80 million years following three functional transfers to the nucleus
Source: BMC Evol Biol. 2006 Jul 14;6:55. doi: 10.1186/1471-2148-6-55 (PMC1543663; doi:10.1186/1471-2148-6-55)
Supplement: Additional File 2 — RPS14 amino acid sequence changes as a result of RNA editing. The table shows the specifics of RNA editing for 15 of the 21 taxa shown in Table 1. cDNA sequences for the other six taxa (Luzula, Oryza, Glyceria, Poa, Bromus, Phragmites; the Oryza cDNA is from Kubo et al. [18] and the Triticum cDNA is from Sandoval et al. [20]) are not shown because they are not RNA-edited. cDNA translations disregard indels, i.e., they assume an intact open reading frame. [file 1471-2148-6-55-S2.pdf]

rps14 cDNAs	Position in Figure 3	RNA edit	Codon position	Pre-edit	Post-edit
Avena, Hordeum, Elymus	5	C → U	2nd	Ser	Leu
Typha	48	U → C	3rd	Leu	Leu
Festuca	50	C → U	2nd	Ala	Val
Elymus	75	C → U	1st	Arg	STOP
Joinvillea	82	U → C	2nd	Leu	Pro
Triticum, Hordeum	108	C → U	3rd	Pro	Pro
Triticum	112	C → U	1st	Leu	Phe
Hordeum, Zeugites	113	U → C	2nd	Leu	Pro
Anomochloa, Elymus, Chasmanthium	118	U → C	1st	Ser	Pro
Hordeum	173	C → U	2nd	Ser	Leu
Juncus	179	C → U	2nd	Ala	Leu
Carex	194	C → U	2nd	Arg	Leu
Typha	208	C → U	2nd	Ser	Phe
Anomochloa	220	C → U	2nd	Pro	Leu
Festuca	222	C → U	1st	Arg	Cys
Bambusa, Secale	225	U → C	1st	Ser	Pro
Hordeum	229	U → C	2nd	Val	Ala
Dendrocalamus	243	C → U	1st	Arg	Cys
Joinvillea, Typha	289	C → U	1st	Pro	Ser
Hordeum	290	C → U	2nd	Ser	Phe
